# Coma

**Published:** 1889-10

**Authors:** J. F. Williams

**Affiliations:** S. C.


### COMA.

By J. F. WILLIAMS, M. D., OF S. C.

EDITORS AND READERS OF THE JOURNAL:—Being young in the profession, and always eager to learn something, I have a case I wish to report to you and get some information, if there is any for me.

Aug. 21st. Was called in haste to see an old lady whom the messenger said had gone to sleep and they could not wake her up.

Upon my arrival I found her in bed soundly asleep, apparently perfectly natural. Upon examination I found pulse 90; temperature 99; Respiration nearly normal. She retired the night before feeling as well as usual, except a little tired, as she had been cutting and drying peaches all day. The family could not tell me whether her bowels had been acting regularly or not. With this information I set about trying to awake her. 1st. I had her plunged in cold water 3 or 4 times and then rubbed her dry with a flannel cloth. 2nd. Used the cold water douche over head and face, all to no avail. 3rd. Aqua ammonia and whiskey was given very freely, an ounce of the former and three or four of the latter, by hypodermic syringe and inhalation. 4th. Blistered her spine, neck and scalp all over. 5th. Bled her a good deal from the arm, and she was just as sound asleep as ever, and is now, this being the 9th day.

I have seen her 3 or 4 times since. Her temperature is about ½ degree higher, pulse about 30 more; now it is 120 per minute; respiration somewhat slower and weaker. I have done all I can for her except the use of an electric battery. I would have used it but she has a very grave chronic heart trouble. I will state that I treated her a year ago for dropsy. Relieved her quickly, and she has not had any since.

Now what is the cause of her sleeping? My opinion is that it is caused by uric acid in the blood, as she has a kidney trouble associated with heart disease, which was the principal cause of the dropsy.

If I am right in my diagnosis I want to know it. I write solely for information.

I wish to know if it would have been advisable to have used the battery.

Brethren, let me hear from you either through the RECORD or by private letter. Give me all the information you can, so I will know how to manage my next case more successfully. Long live the So. MED. RECORD. Any further information will be furnished if desired by any one.

[Without time to investigate carefully the above case, and in the absence of urinary tests and other facts which a close inquiry and careful examination of the patient might furnish for a more certain diagnosis, we incline to the opinion that the case is one of *uræmic coma*, dependent upon Bright's Disease, or other kidney trouble.

We should be pleased to publish in the RECORD the views of medical brethren in regard to the above case.—ED. RECORD.]

